# Supplementary material for: Cell barrier function of resident peritoneal macrophages in post-operative adhesions
Source: Nat Commun. 2021 Apr 14;12:2232. doi: 10.1038/s41467-021-22536-y (PMC8046819; doi:10.1038/s41467-021-22536-y)
Supplement: Supplementary file 1 — Supplementary Information [file 41467_2021_22536_MOESM1_ESM.pdf]

Supplementary Information

Cell barrier function of resident peritoneal macrophages in post-operative adhesions

Authors:

Tomoya Ito[1], Yusuke Shintani[1], Laura Fields[1], Manabu Shiraishi[1], Mihai-Nicolae Podaru[1], Satoshi Kainuma[1], Kizuku Yamashita[1], Kazuya Kobayashi[1], Mauro Perretti[1], Fiona Lewis-McDougall[1], Ken Suzuki[1]

Affiliations:

[1]William Harvey Research Institute, Barts and The London School of Medicine and Dentistry, Queen Mary University of London, United Kingdom

Supplementary information includes:

- Supplementary Fig. 1 to 14
- Supplementary Table 1, 2

Supplementary Fig. 1

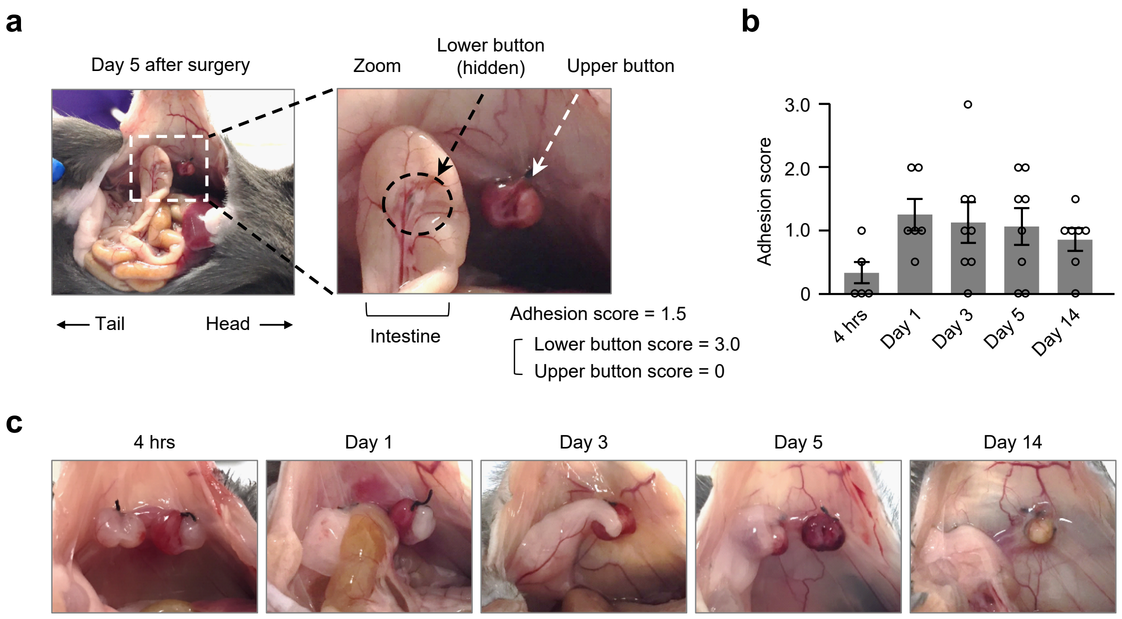

Supplementary Fig. 1: Post-operative adhesions are formed as early as day 1 post-surgery in the mouse ischemic button model.

(**a**) Representative image of an abdominal adhesion on day 5 post-ischemic button creation in mice. Black and white dashed arrows show the lower and upper ischemic button, respectively.

(**b**, **c**) Time course of the average adhesion score (**b**) and representative images of abdominal adhesions (**c**) after ischemic button creation. *n* = 6 (4 hrs), 6 (Day 1), 8 (Day 3), 8 (Day 5) and 7 (Day 14) mice. Data are shown as the mean ± SEM.

Supplementary Fig. 2

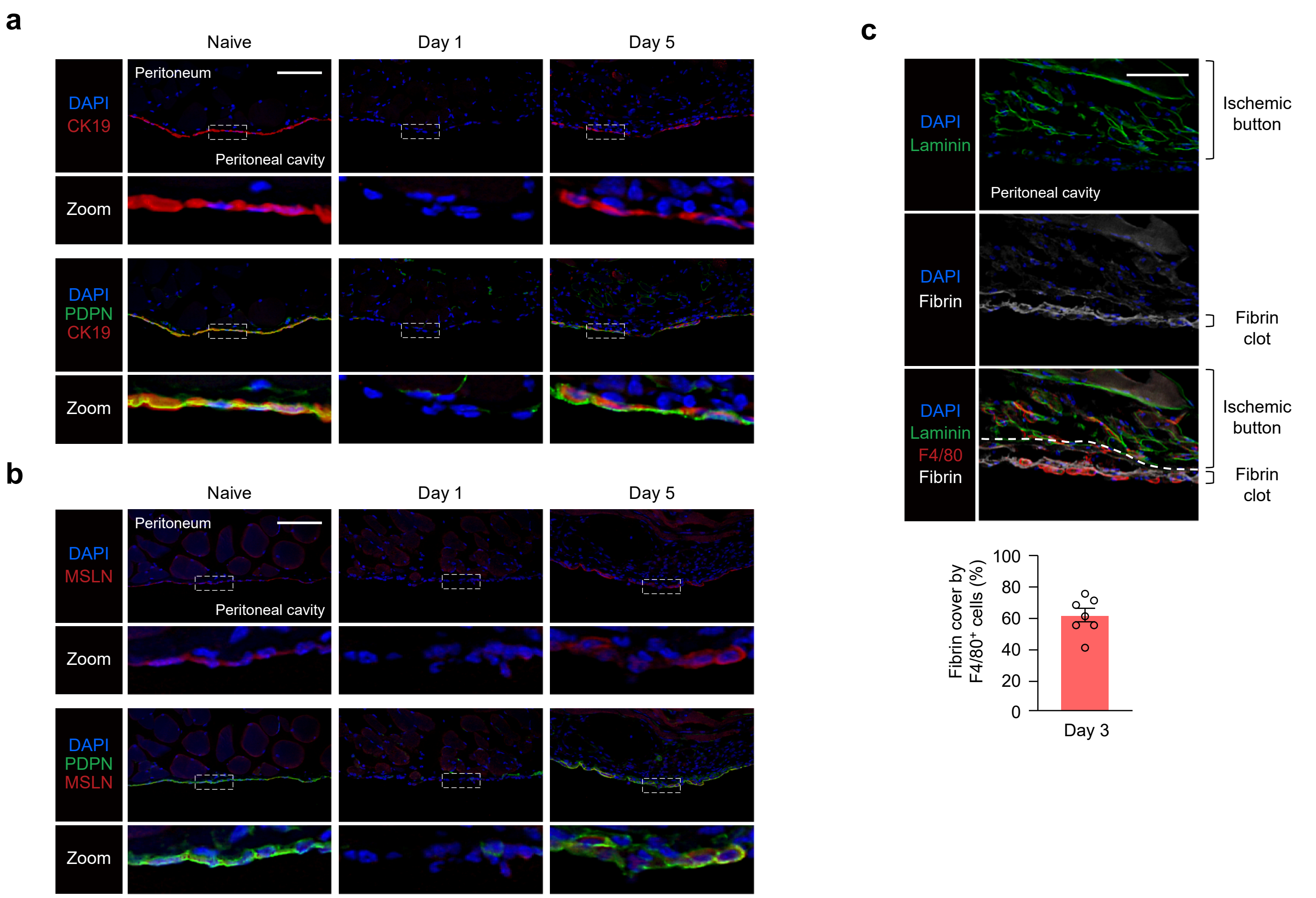

Supplementary Fig. 2: The mesothelial cell layer is disrupted and fibrin clots are formed on the damaged peritoneum post-surgery.

(**a**, **b**) Representative images of immunofluorescence staining of the ischemic button using the anti-cytokeratin 19 (CK19) antibody (**a**) and anti-mesothelin (MSLN) antibody (**b**) as mesothelial markers. The naive peritoneum was used as control. n = 3 independent mice. Scale bars, 100 µm.

(**c**) Representative immunofluorescence images and quantification of the coverage of fibrin clots by F4/80^{+} cells on the ischemic button on day 3 post-surgery. The anti-laminin antibody was to identify the basement membrane. The white dashed line represents the border between the ischemic button and fibrin clot. Scale bar, 100 µm. n = 7 mice. Data are shown as the mean ± SEM.

Supplementary Fig. 3

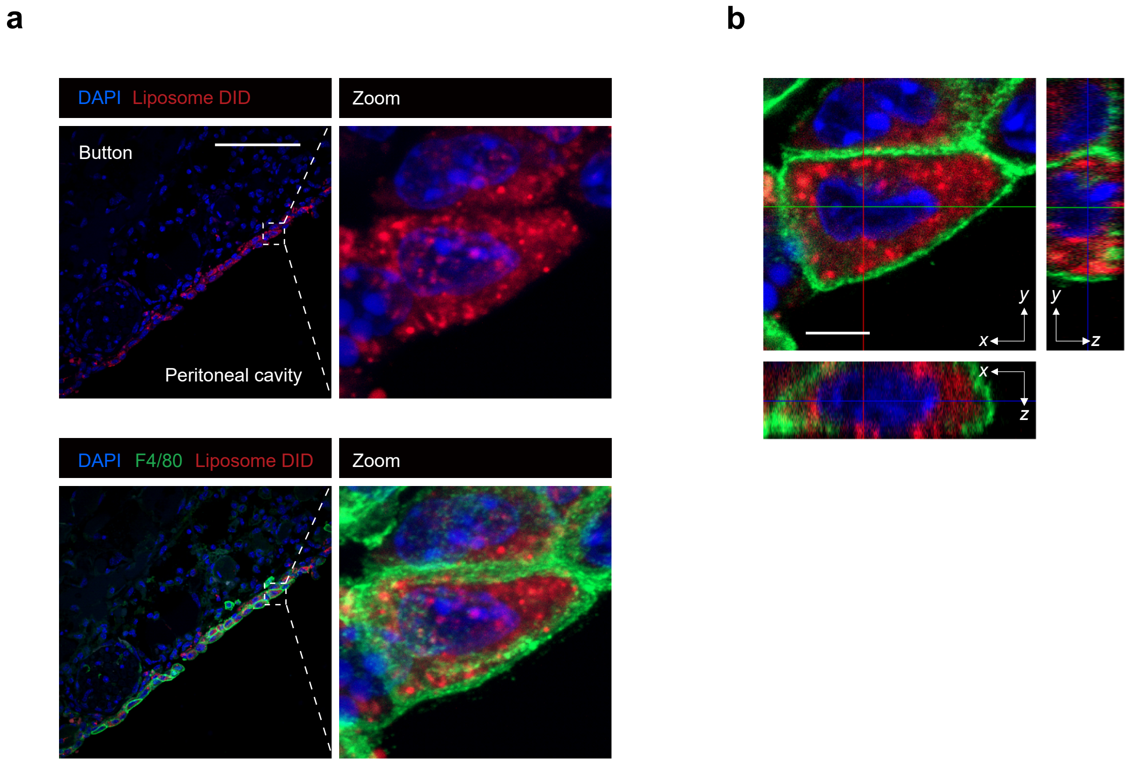

Supplementary Fig. 3: Accumulated macrophages on the ischemic button exhibit liposome uptake. (**a, b**) Representative confocal images of the maximum intensity projection (**a**) and the orthogonal view (**b**) of the ischemic button on day 3 after surgery. Liposome DID was injected intraperitoneally on day 2 post-ischemic button creation-surgery. Cross sections were stained for F4/80. Confocal images were captured in z-stacks and reconstructed with the maximum intensity projection (stack of 9 pictures of 0.5 µm) and orthogonal view (stack of 29 pictures of 0.21 µm). $n = 3$ independent ischemic buttons. Scale bars, 100 µm (**a**) and 5 µm (**b**), respectively.

Supplementary Fig. 4

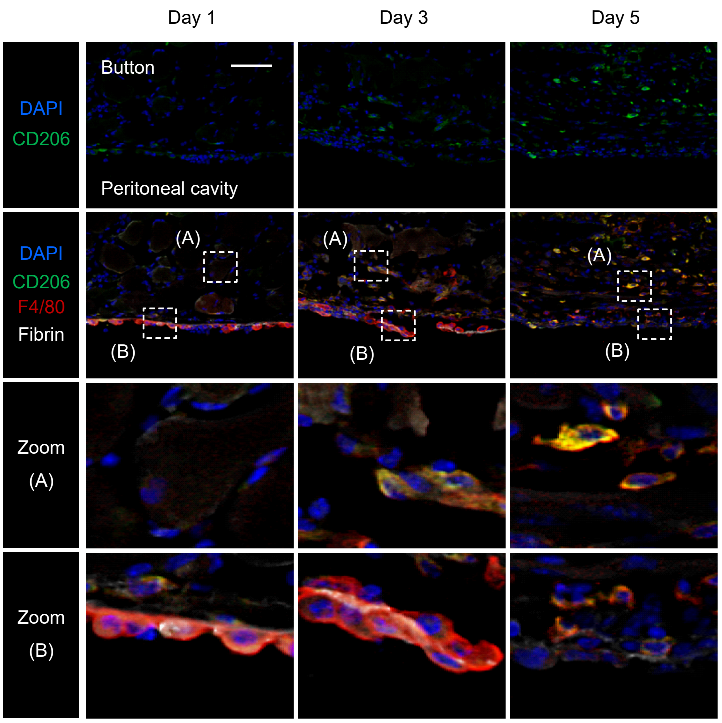

Supplementary Fig. 4: CD206⁺ macrophages localize within the ischemic button tissue after day 3. Representative immunofluorescence images of ischemic buttons created in the mouse peritoneal membrane. Cross sections were stained for CD206, F4/80 and fibrin. Zooms (A) and (B) present the higher magnification images of the inside and surface of the ischemic button, respectively. $n = 3$ independent ischemic buttons for each time point. Scale bars, 100 µm.

Supplementary Fig. 5

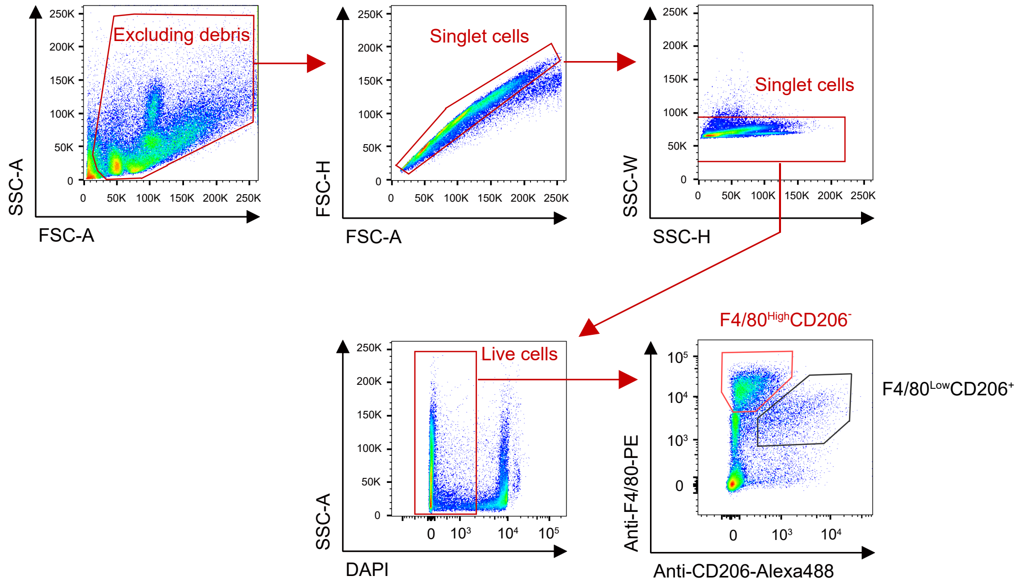

Fig. 3a (Day 5)

Supplementary Fig. 5: The gating strategy is optimized to identify two peritoneal macrophage subsets by flow cytometry.

Exemplifying the gating strategy for flow cytometry analysis of the peritoneal macrophages. Forward versus side scatter (FSC-A vs SSC-A) gating was used to identify cells of interest and exclude the debris. Singlets were gated according to the pattern of FSC-A versus FSC-H, followed by SSC-H versus SSC-W. Dead cells were excluded by DAPI staining.

Supplementary Fig. 6

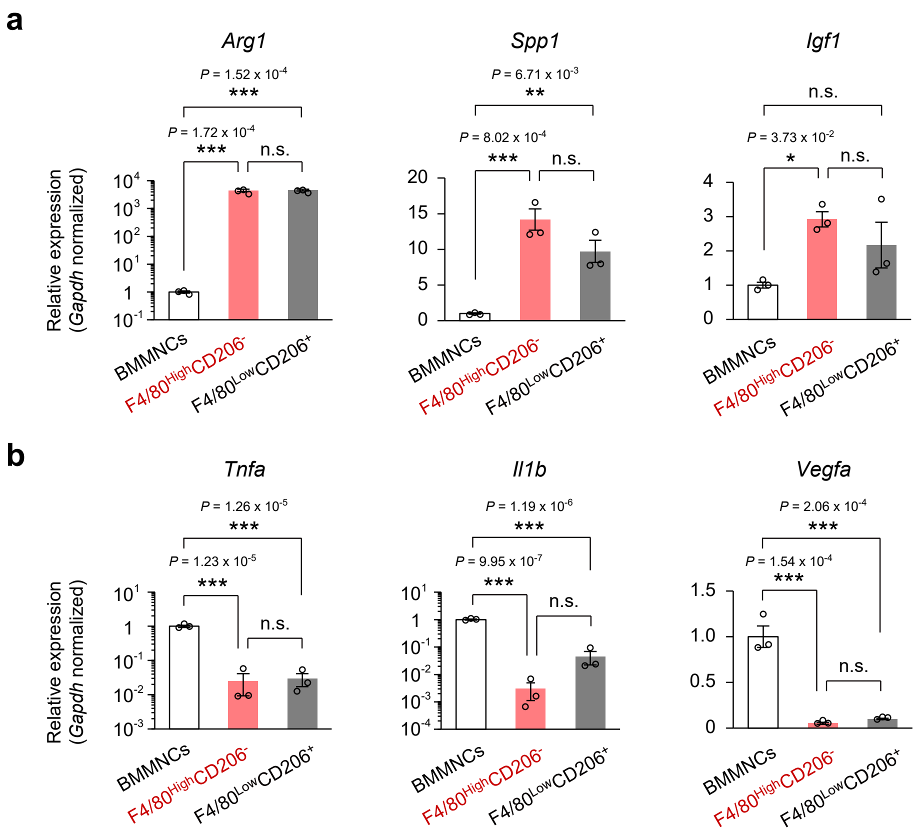

Supplementary Fig. 6: Peritoneal macrophage subsets show similar expression patterns of pro-/anti-inflammatory genes.

(**a, b**) Quantitative RT-PCR analysis for expression of pro-/anti-inflammatory genes. F4/80^{High}CD206^{-} (red) and F4/80^{Low}CD206^{+} (grey) macrophages were FACS-sorted from the peritoneal cavity on day 5 after surgery and assessed. Bone marrow mononuclear cells (BMMNCs) were used as control. *n* = 3 mice per group. Data are shown as the mean ± SEM. **P* < 0.05, ***P* < 0.01, ****P* < 0.001, one-way ANOVA and Tukey's post hoc test.

Supplementary Fig. 7

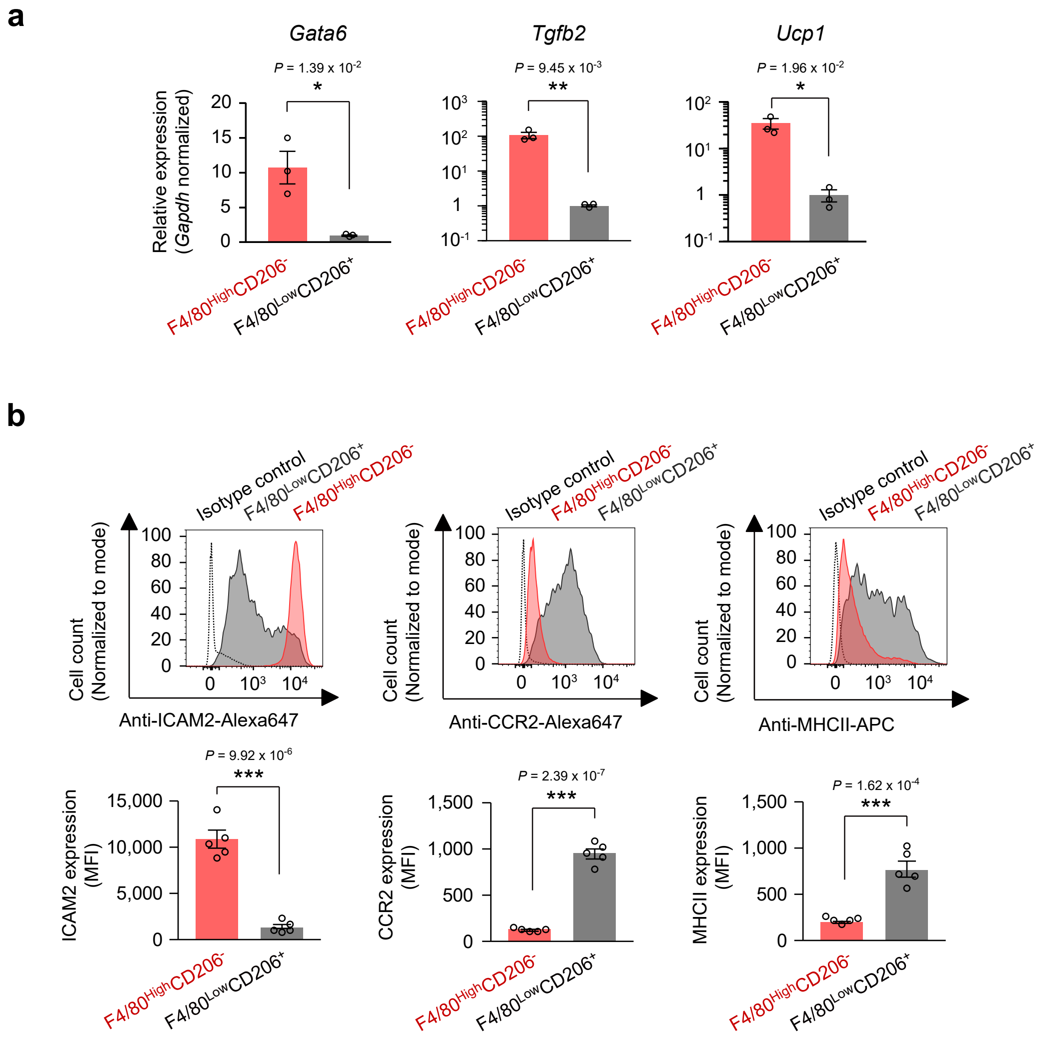

Supplementary Fig. 7: Peritoneal macrophage subsets exhibit different expression patterns of macrophage subset marker genes/proteins.

(**a**) Quantitative RT-PCR analysis for expression of peritoneal macrophage marker genes. F4/80^{High}CD206^{-} (red) and F4/80^{Low}CD206^{+} (grey) macrophages were FACS-sorted from the peritoneal cavity on day 5 after surgery and assessed. *n* = 3 mice per group.

(**b**) Representative flow cytometry histograms showing expression of ICAM2, CCR2 and MHCII of two macrophage subsets on day 5 after surgery. Quantification of median fluorescence intensity (MFI) values is shown in the lower panels. *n* = 5 mice.

Data are shown as the mean ± SEM. **P* < 0.05, ***P* < 0.01, ****P* < 0.001, two-tailed Student's *t*-test.

Supplementary Fig. 8

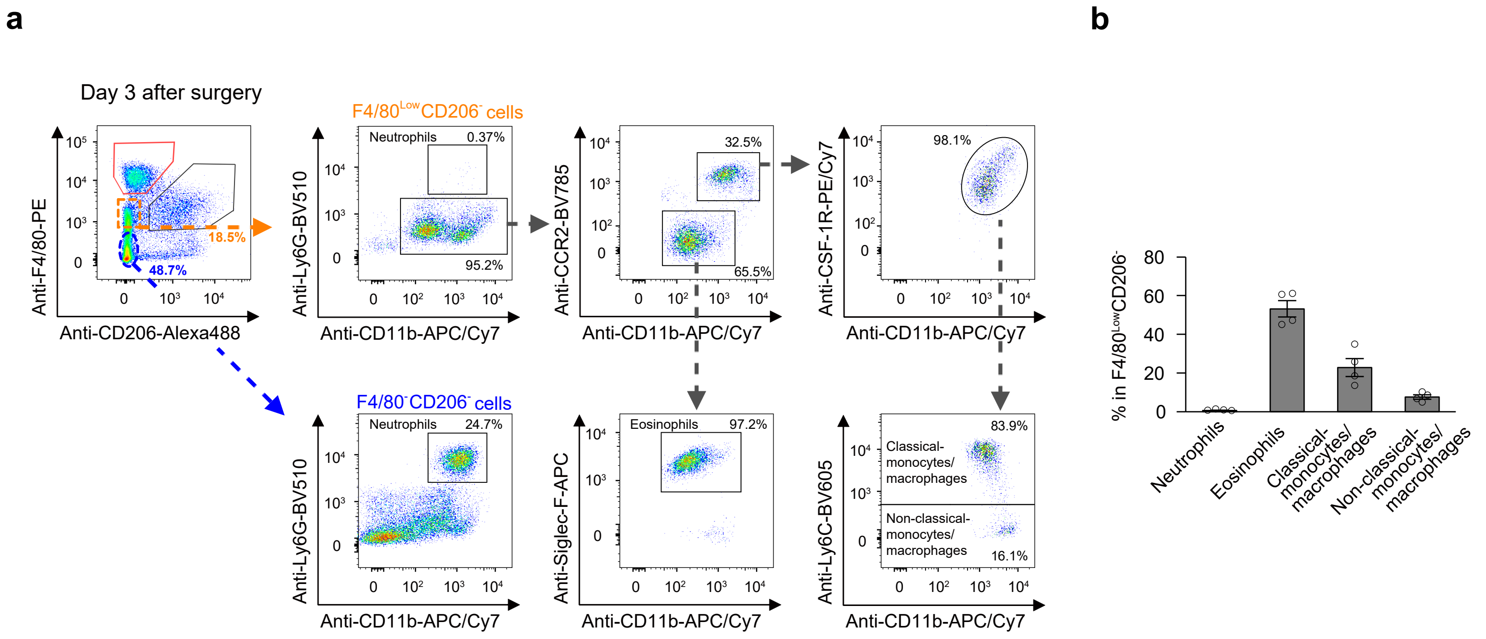

Supplementary Fig. 8: F4/80^{Low}CD206^{-} cells in peritoneal cavity after surgery include multiple cell types. (**a, b**) Exemplifying the gating strategy and quantification of different immune cells in the peritoneal cavity on day 3 after surgery. The bar graph represents the percentage of neutrophils, eosinophils, classical-monocytes/macrophages and non-classical-monocytes/macrophages in F4/80^{Low}CD206^{-} cells (**b**). n = 4 mice per group. Data represent the mean ± SEM.

Supplementary Fig. 9

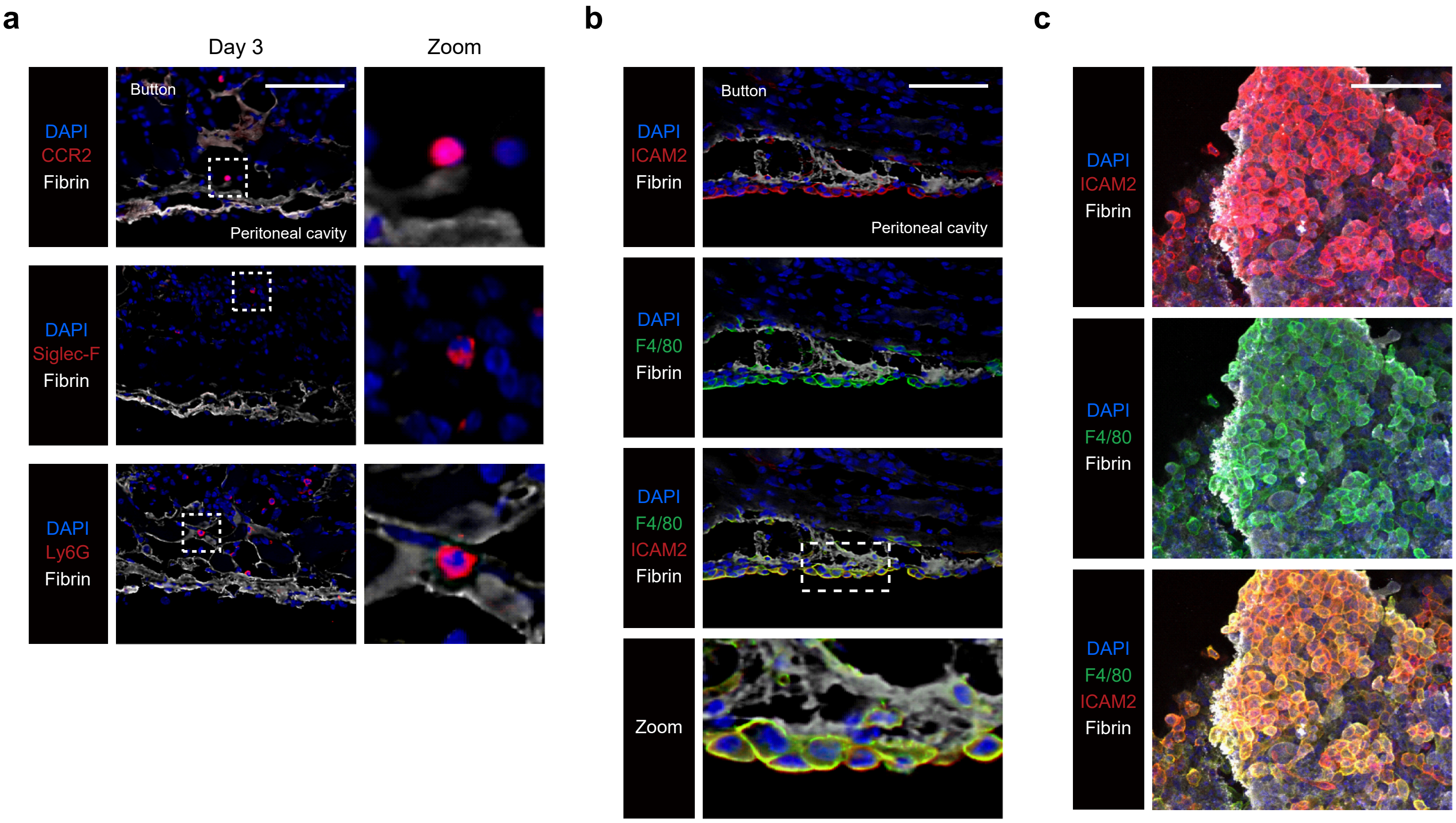

Supplementary Fig. 9: Resident peritoneal macrophage subset specifically covers the fibrin clots.

(**a**) Representative images of immunofluorescence staining of the ischemic button on day 3 post-surgery. The CCR2 antibody, Siglec-F antibody and Ly6G antibody were used as recruited macrophage, eosinophil and neutrophil markers, respectively.

(**b, c**) Representative images of immunofluorescence staining of cross section (**b**) and whole-mount staining (**c**) of the ischemic button surface on day 3 post-surgery. The ischemic button was stained with ICAM2, F4/80 and Fibrin antibodies.

n = 3 independent mice. Scale bars, 100 µm

Supplementary Fig. 10

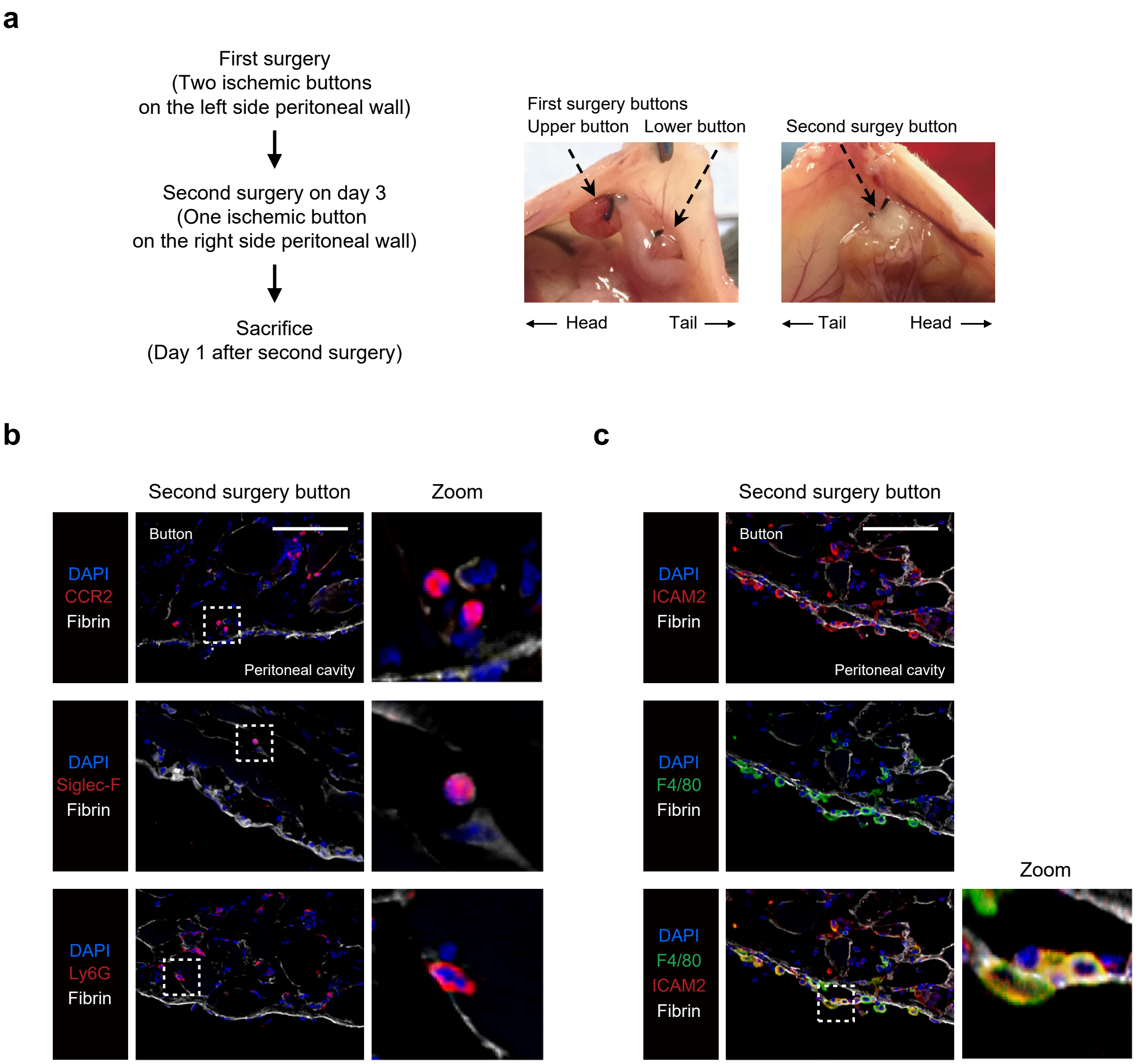

Supplementary Fig. 10: Recruited macrophages, eosinophils or neutrophils do not show a capability to cover the fibrin clots.

(**a**) Schematic of the repeated ischemic button creation experiment and representative images of the ischemic buttons after second surgery.

(**b, c**) Representative images of immunofluorescence staining of the ischemic button on day 1 after second surgery. The CCR2 antibody, Siglec-F antibody and Ly6G antibody were used as recruited macrophage, eosinophil and neutrophil markers, respectively (**b**). The ischemic button was co-stained with ICAM2, F4/80 and Fibrin antibodies (**c**). *n* = 3 independent mice. Scale bars, 100 µm

Supplementary Fig. 11

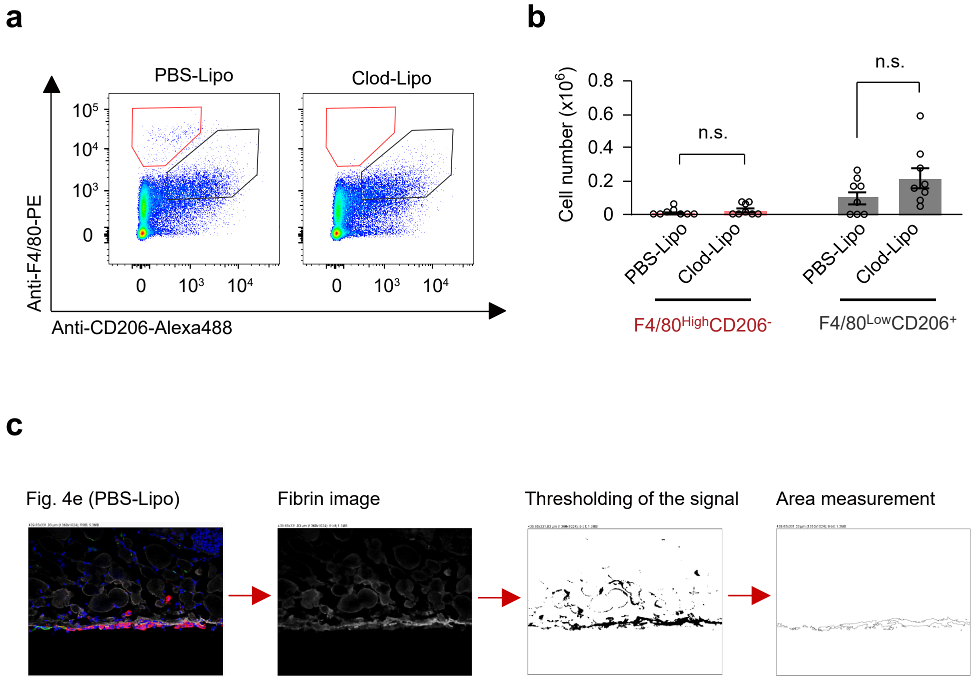

Supplementary Fig. 11: Clodronate liposome injection depletes resident F4/80^{High}CD206^{-} peritoneal macrophages. (**a**, **b**) Representative flow cytometry plots (**a**) and absolute cell numbers (**b**) of F4/80^{High}CD206^{-} macrophages (red) and F4/80^{Low}CD206^{+} (black) macrophages in the peritoneal fluid on day 1 post-ischemic button creation.
n = 8 mice in each group. Data are shown as the mean ± SEM. n.s. not significant, two-tailed Student's *t*-test.
(**c**) Schematic representation of the quantification strategy of fibrin formation.

Supplementary Fig. 12

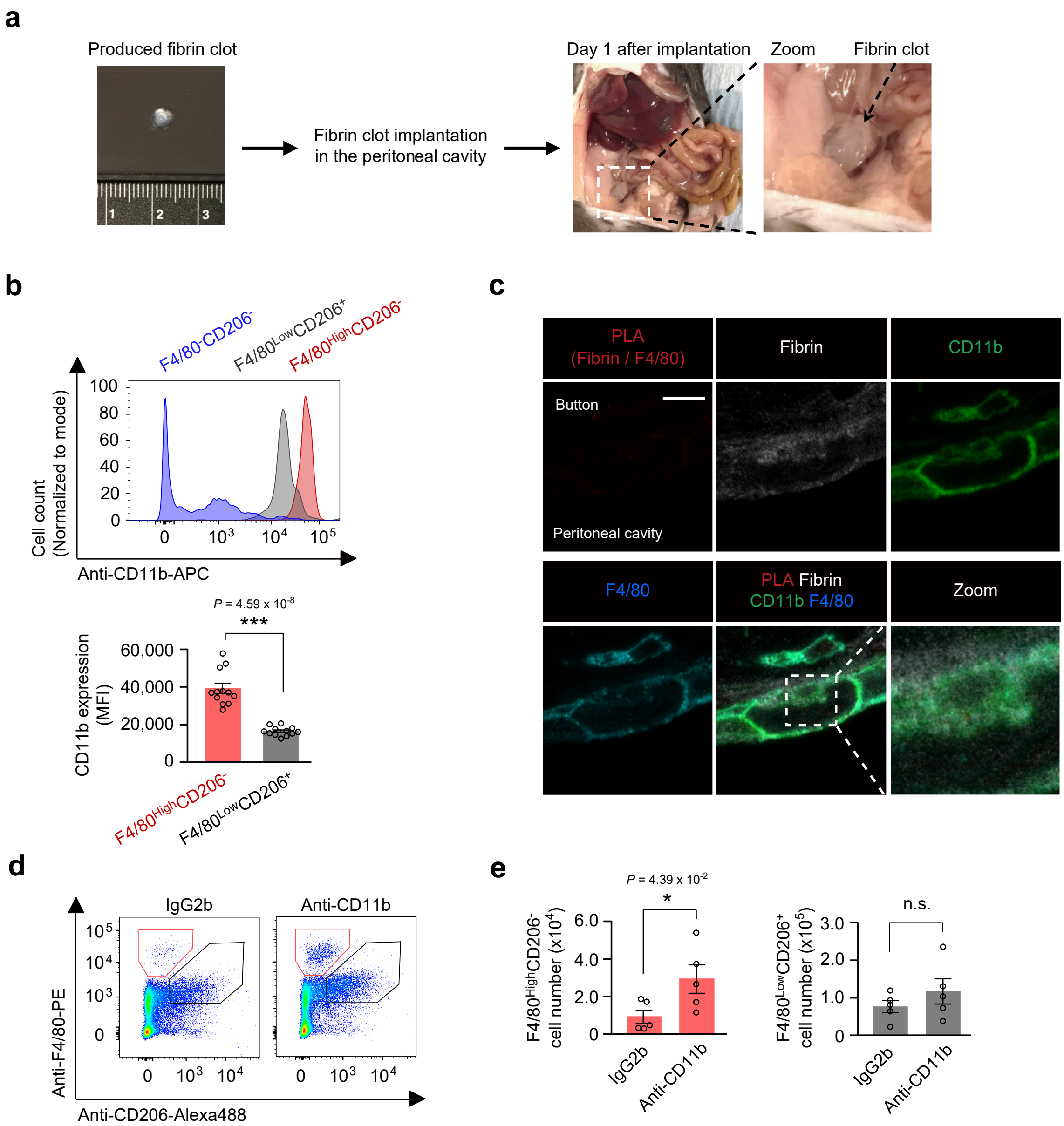

Supplementary Fig. 12: Resident F4/80^{High}CD206^{-} peritoneal macrophages accumulate on the fibrin surface through their expression of CD11b.

(**a**) Scheme of the experiment of intraperitoneal implantation of exogenous fibrin clot.

(**b**) Representative flow cytometry histogram showing CD11b expression of F4/80^{-}CD206^{-} peritoneal cells (blue), resident F4/80^{High}CD206^{-} peritoneal macrophages (red) and recruited F4/80^{Low}CD206^{+} peritoneal macrophages (grey) in the peritoneal cavity on day 5 after surgery. Quantification of the median fluorescence intensity (MFI) values is shown in the bar graph. n = 12 mice. Data are shown as the mean ± SEM. ***P < 0.001, t-test.

(**c**) Representative images of in situ proximity ligation assay (PLA) (red) between fibrin and F4/80. Scale bars, 5 µm. n = 3 independent experiments.

(**d**, **e**) Representative flow cytometry plots (**d**) and absolute cell numbers (**e**) of F4/80^{High}CD206^{-} macrophages (red) and F4/80^{Low}CD206^{+} macrophages (grey) in the peritoneal cavity treated with anti-CD11b (5C6) antibody or rat IgG2b control antibody on day 1 post-ischemic button creation. n = 7 (IgG2b group) and 6 (Anti-CD11b group) mice.

Data represent the mean ± SEM. *P < 0.05, ***P < 0.001, n.s. not significant, t-test.

Supplementary Fig. 13

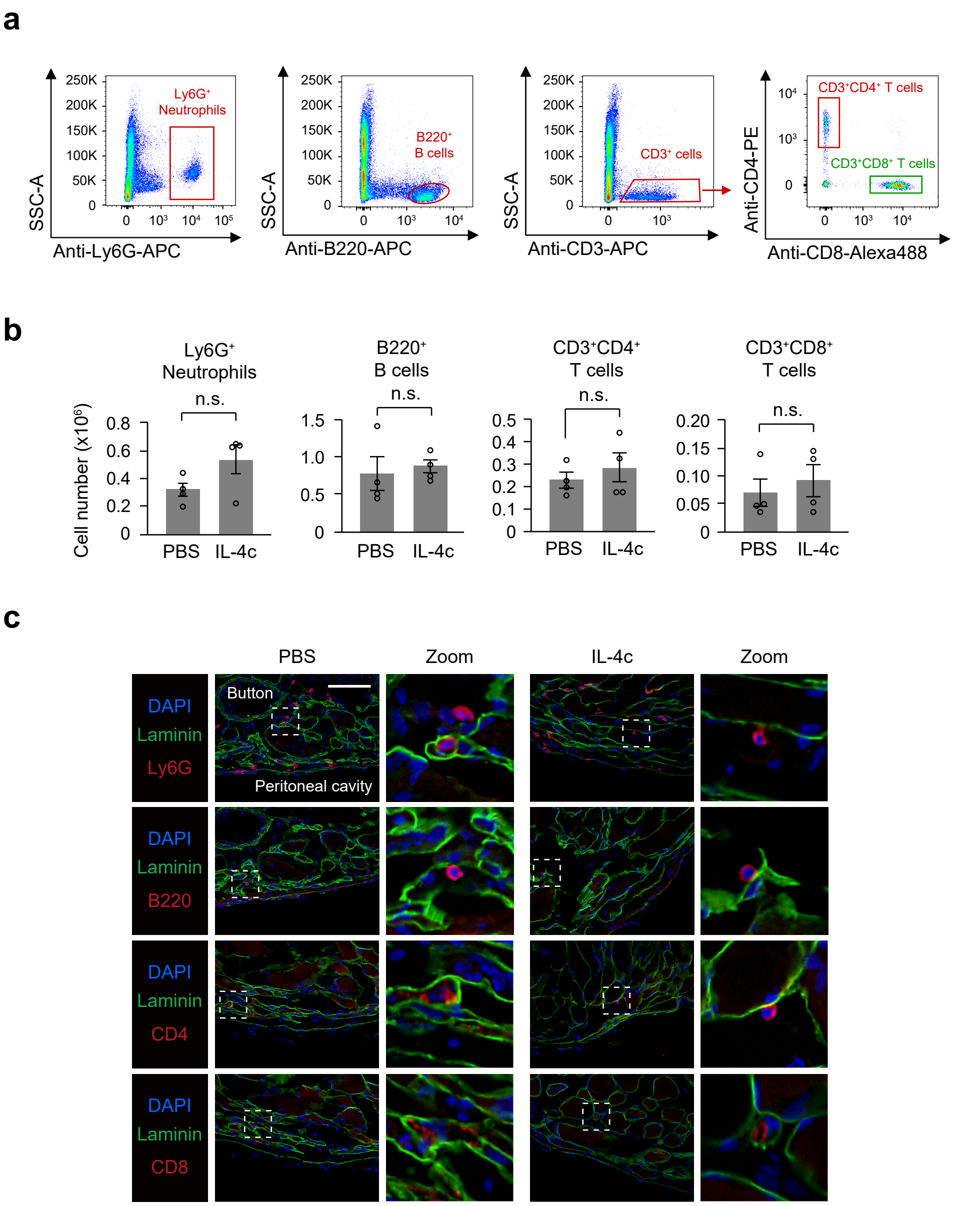

Supplementary Fig. 13: IL-4c administration does not affect the numbers of other immune cells in the peritoneal fluid or within the ischemic button.

(**a**, **b**) Exemplifying the gating strategy (**a**) and absolute cell numbers (**b**) of Ly6G$^+$ neutrophils, B220$^+$ B lymphocytes, CD3$^+$CD4$^+$ and CD3$^+$CD8$^+$ T lymphocytes in the peritoneal cavity on day 3 after ischemic button creation with intraperitoneal administration of IL-4c or PBS. $n = 4$ mice per group. Data represent the mean ± SEM. n.s. not significant, *t*-test.

(**c**) Representative images of immunofluorescence staining of the ischemic button on day 3 after surgery with intraperitoneal administration of IL-4c or PBS. The anti-laminin antibody was used to detect the basement membrane. $n = 3$ mice in each group. Scale bars, 100 µm.

Supplementary Fig. 14

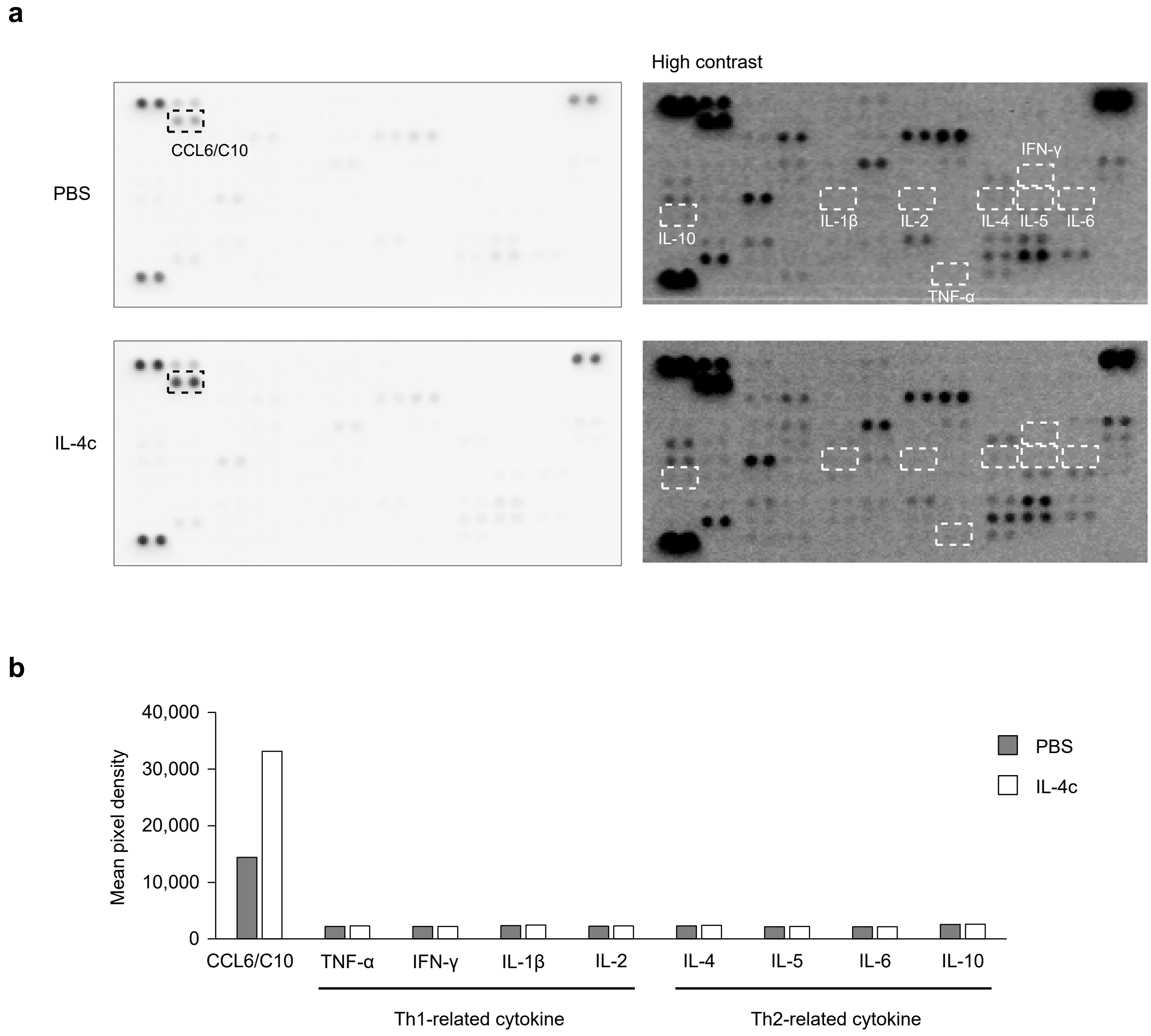

Supplementary Fig. 14: IL-4c administration does not affect the Th1/Th2-related cytokine profile in the peritoneal fluid after surgery.

(**a**) Representative images of the mouse cytokine antibody array showing the reactivity of peritoneal fluid samples. Peritoneal fluid containing 20 μg protein was pooled from 6 mice on day 3 after ischemic button creation with intraperitoneal administration of IL-4c or PBS. Right panels show high contrast images. White and black boxes indicate the location of the representative Th1/Th2-related cytokines and CCL6/C10, a representative peritoneal macrophage-derived chemokine, respectively.

(**b**) The quantitative analysis of the pixel intensity data. The average mean fluorescence intensity data for all cytokines are available in Source Data.

Supplementary Table 1. A list of antibodies used in this study

Antibodies	Companies	Catalogues / Identifiers
Antibodies for immunohistochemistry and whole-mount staining:		
Anti-F4/80 (clone: BM8), 1:200 dilution	eBioscience	Cat# 14-4801-81<br>RRID: AB_467557
Anti-F4/80 (clone: BM8), biotin conjugated, 1:200 dilution	BioLegend	Cat# 123105<br>RRID: AB_893499
Anti-Fibrin, 1:500 dilution	Abcam	Cat# ab34269<br>RRID: AB_732367
Anti-PDPN (clone: 8.1.1), 1:1000 dilution	eBioscience	Cat# 14-5381-81<br>RRID: AB_1210506
Anti-cytokeratin 19 (clone: EP1580Y), 1:500 dilution	Abcam	Cat# ab52625<br>RRID: AB_2281020
Anti-mesothelin, 1:100 dilution	Invitrogen	Cat# PA5-79698<br>RRID: AB_2746813
Anti-laminin, 1:1000 dilution	Sigma	Cat# L9393<br>RRID: AB_477163
Anti-CD206 (clone: C068C2), 1:200 dilution	BioLegend	Cat# 141702<br>RRID: AB_10900233
Anti-CCR2 (clone: SA203G11), Alexa Fluor 647 conjugated, 1:100 dilution	BioLegend	Cat# 150603<br>RRID: AB_2566139
Anti-Siglec-F (clone: S17007L), APC conjugated, 1:100 dilution	BioLegend	Cat# 155507<br>RRID: AB_2750236
Anti-Ly6G (clone: 1A8), APC conjugated, 1:100 dilution	BioLegend	Cat# 127613<br>RRID: AB_1877163
Anti-ICAM2 (CD102) (clone: 3C4), Alexa Fluor 647 conjugated, 1:100 dilution	BioLegend	Cat# 105611<br>RRID: AB_2122183
Anti-B220 (clone: RA3-6B2), biotin conjugated, 1:200 dilution	eBioscience	Cat# 13-0452-82<br>RRID: AB_466449
Anti-CD4 (clone: H129.19), biotin conjugated, 1:200 dilution	BD Biosciences	Cat# 553648<br>RRID: AB_394968
Anti-CD8a (clone: 53-6.7), 1:200 dilution	BD Biosciences	Cat# 558733<br>RRID: AB_397092
Antibodies for flow cytometry:		
Anti-mouse CD16/CD32 (clone: 93), 1:100 dilution	Invitrogen	Cat# 14-0161-85<br>RRID: AB_467134
Anti-CD206 (clone: C068C2), Alexa Fluor 488 conjugated, 1:100 dilution	BioLegend	Cat# 141710<br>RRID: AB_10900445
Anti-F4/80 (clone: BM8), PE conjugated, 1:200 dilution	BioLegend	Cat# 123110<br>RRID: AB_893486
Anti-CD11b (clone: M1/70), APC conjugated, 1:400 dilution	eBioscience	Cat# 17-0112-82<br>RRID: AB_469343
Anti-CCR2 (clone: SA203G11), Alexa Fluor 647 conjugated, 1:100 dilution	BioLegend	Cat# 150603<br>RRID: AB_2566139

Anti-MHCII (I-Ab) (clone: AF6-120.1), APC conjugated, 1:200 dilution	BioLegend	Cat# 116417<br>RRID: AB_10575761
Anti-ICAM2 (CD102) (clone: 3C4), Alexa Fluor 647 conjugated, 1:200 dilution	BioLegend	Cat# 105611<br>RRID: AB_2122183
Anti-CCR2 (clone: SA203G11), Brilliant Violet 785 conjugated, 1:100 dilution	BioLegend	Cat# 150621<br>RRID: AB_2721565
Anti-CD11b (clone: M1/70), APC/Cyanine7 conjugated, 1:400 dilution	BioLegend	Cat# 101226<br>RRID: AB_830642
Anti-CSF-1R (clone: AFS98), PE/Cyanine7 conjugated, 1:200 dilution	BioLegend	Cat# 135523<br>RRID: AB_2566459
Anti-Ly6C (clone: HK1.4), Brilliant Violet 605 conjugated, 1:200 dilution	BioLegend	Cat# 128035<br>RRID: AB_2562352
Anti-Siglec-F (clone: S17007L), APC conjugated, 1:100 dilution	BioLegend	Cat# 155507<br>RRID: AB_2750236
Anti-Ly6G (clone: 1A8), Brilliant Violet 510 conjugated, 1:200 dilution	BioLegend	Cat# 127633<br>RRID: AB_2562937
Anti-Ly6G (clone: 1A8), APC conjugated, 1:200 dilution	BioLegend	Cat# 127613<br>RRID: AB_1877163
Anti-B220 (clone: RA3-6B2), APC conjugated, 1:200 dilution	BioLegend	Cat# 103211<br>RRID: AB_312996
Anti-mouse CD3ε (clone: 145-2C11), APC conjugated, 1:200 dilution	BioLegend	Cat# 100311<br>RRID: AB_312676
Anti-CD4 (clone: H129.19), PE conjugated, 1:200 dilution	BD Biosciences	Cat# 553652<br>RRID: AB_394972
Anti-CD8a (clone: 53-6.7), Alexa Fluor 488 conjugated, 1:200 dilution	BioLegend	Cat# 100726<br>RRID: AB_493423
Rat IgG2a kappa Isotype Control, Alexa Fluor 488 conjugated	AbD Serotec	Cat# MCA1124A488<br>RRID: AB_567359
Rat IgG2a kappa Isotype Control (eBR2a), PE conjugated	eBioscience	Cat# 12-4321-41<br>RRID: AB_1518774
Rat IgG2a kappa Isotype Control (RTK2758), APC conjugated	BioLegend	Cat# 400511
Rat IgG2b kappa Isotype Control (eB149/10H5), APC conjugated	eBioscience	Cat# 17-4031-81<br>RRID: AB_470175
Rat IgG2a kappa Isotype Control (RTK2758), Alexa Fluor 647 conjugated	BioLegend	Cat# 400526
Rat IgG2b kappa Isotype Control (RTK4530), Alexa Fluor 647 conjugated	BioLegend	Cat# 400626<br>RRID: AB_389343
Rat IgG2b kappa Isotype Control (RTK4530), Brilliant Violet 785 conjugated	BioLegend	Cat# 400647
Rat IgG2b kappa Isotype Control (RTK4530), APC/Cyanine7 conjugated	BioLegend	Cat# 400623<br>RRID: AB_326565
Rat IgG2a kappa Isotype Control (RTK2758), PE/Cyanine7 conjugated	BioLegend	Cat# 400521<br>RRID: AB_326542
Rat IgG2c kappa Isotype Control (RTK4174), Brilliant Violet 605 conjugated	BioLegend	Cat# 400727
Rat IgG2a kappa Isotype Control (RTK2758), Brilliant Violet 510 conjugated	BioLegend	Cat# 400547

Antibodies for Proximity ligation assay:		
Anti-CD11b (clone: M1/70), 1:200 dilution	eBioscience	Cat# 14-0112-85<br>RRID: AB_467109
Anti-Fibrin, 1:500 dilution	Abcam	Cat# ab34269<br>RRID: AB_732367
Goat anti-Rat IgG, AlexaFluor 488 conjugated, 1:300 dilution	Invitrogen	Cat# A11006<br>RRID: AB_2534074
Anti-F4/80 (clone: BM8), APC conjugated, 1:200 dilution	BioLegend	Cat# 123116<br>RRID: AB_893481
Goat anti-Rabbit IgG, AlexaFluor 405 conjugated, 1:300 dilution	Invitrogen	Cat# A31556<br>RRID: AB_221605
Anti-F4/80 (clone: BM8), 1:200 dilution	eBioscience	Cat# 14-4801-81<br>RRID: AB_467557
Anti-CD11b (clone: M1/70), APC conjugated, 1:200 dilution	eBioscience	Cat# 17-0112-82<br>RRID: AB_469343
Antibodies for in vivo treatment:		
Anti-CD11b (clone: 5C6)	Invitrogen	Cat# MA5-16528<br>RRID: AB_2538033
Rat IgG2b kappa Isotype Control (eB149/10H5)	eBioscience	Cat# 14-4031-85<br>RRID: AB_470100
Anti-IL-4 (clone: BVD4-1D11)	BD Biosciences	Cat# 554387<br>RRID: AB_398545

Supplementary Table 2. A list of primer sequences used in this study

Gene	Forward primer (5′ to 3′)	Reverse primer (5′ to 3′)
Arg1	CAAGACAGGGCTCCTTTCAG	AAGCAAGCCAAGGTTAAAGC
Spp1	GATAGCTTGGCTTATGGACTGAGGT	GACTCCTTAGACTCACCGCTCTT
Igf1	GGACCGAGGGGCTTTTACTTC	GGCACAGTACATCTCCAGTCTCCTC
Tnfa	ATGGCCTCCCTCTCATCAGTTC	TCTTTGAGATCCATGCCGTTG
Il1b	CAAGCAACGACAAAATACCTGTG	ACCGTTTTTCCATCTTCTTCTTTGG
Vegfa	GAGGCAGCTTGAGTTAAACGAAC	GTGACATGGTTAATCGGTCTTTCC
Gata6	GACTGCACCAAAAATACTTCTCCTTCT	AGGCCGTCTTGACCTGAATACTTG
Tgfb2	CCACATCTCCTGCTAATGTTGTTG	TAAAATCAATGTAAAGAGGGCGAAGG
Ucp1	GGGCATTCAGAGGCAAATCAG	GAACACTGCCACACCTCCAGT
Gapdh	TAGACAAAATGGTGAAGGTCGGTGT	AATGAAGGGGTCGTTGATGG
