# Supplementary material for: Cell barrier function of resident peritoneal macrophages in post-operative adhesions
Source: Nat Commun. 2021 Apr 14;12:2232. doi: 10.1038/s41467-021-22536-y (PMC8046819; doi:10.1038/s41467-021-22536-y)
Supplement: Supplementary file 3 — Reporting Summary [file 41467_2021_22536_MOESM3_ESM.pdf]

nature research

Corresponding author(s): Ken Suzuki

Last updated by author(s): Feb 6, 2021

Reporting Summary

Nature Research wishes to improve the reproducibility of the work that we publish. This form provides structure for consistency and transparency in reporting. For further information on Nature Research policies, see Authors & Referees and the Editorial Policy Checklist .

Statistics

For all statistical analyses, confirm that the following items are present in the figure legend, table legend, main text, or Methods section.

n/a	Confirmed	
☐	☒	The exact sample size (*n*) for each experimental group/condition, given as a discrete number and unit of measurement
☐	☒	A statement on whether measurements were taken from distinct samples or whether the same sample was measured repeatedly
☐	☒	The statistical test(s) used AND whether they are one- or two-sided<br>*Only common tests should be described solely by name; describe more complex techniques in the Methods section.*
☒	☐	A description of all covariates tested
☐	☒	A description of any assumptions or corrections, such as tests of normality and adjustment for multiple comparisons
☐	☒	A full description of the statistical parameters including central tendency (e.g. means) or other basic estimates (e.g. regression coefficient) AND variation (e.g. standard deviation) or associated estimates of uncertainty (e.g. confidence intervals)
☐	☒	For null hypothesis testing, the test statistic (e.g. *F*, *t*, *r*) with confidence intervals, effect sizes, degrees of freedom and *P* value noted<br>*Give P values as exact values whenever suitable.*
☒	☐	For Bayesian analysis, information on the choice of priors and Markov chain Monte Carlo settings
☒	☐	For hierarchical and complex designs, identification of the appropriate level for tests and full reporting of outcomes
☒	☐	Estimates of effect sizes (e.g. Cohen's *d*, Pearson's *r*), indicating how they were calculated

Our web collection on statistics for biologists contains articles on many of the points above.

Software and code

Policy information about availability of computer code

Data collection	Flow cytometric analyses were performed using BD LSRFortessa cell analyzer (BD Biosciences).<br>Cell sorting was performed using a FACS Aria II (BD Biosciences).<br>Microscopic pictures were acquired by All-in-One microscope (BZ-8000; KEYENCE) or inverted Zeiss 800 confocal microscope (Carl Zeiss).<br>Cell number was assessed using a Countess II Automated Cell Counter (Invitrogen, AMQAX1000).<br>ELISA and Protein concentrations were measured using the SPECTRA MR Microplate Spectrophotometer (Dynex Technologies).<br>RNA concentration and quality were measured using the DS-11 Spectrophotometer (DeNovix).<br>Real-time PCR was performed by Quant studio 7 qPCR machine (Applied Biosystems).<br>Antibody array was measured using the Azure c600 Imager (Azure Biosystems).
Data analysis	Flow cytometric data were analysed by FlowJo software version 10 (Tree Star).<br>Microscopy pictures were analysed by ZEN black 3.0 lite (Carl Zeiss), BZ image analyzer (KEYENCE) and ImageJ software (NIH).<br>Real-time PCR data was analysed by QuantStudio Software v1.3 (Applied Biosystems).<br>All statistical tests were performed using R statistical software (R Foundation) and GraphPad Prism 8 software (GraphPad Inc).

For manuscripts utilizing custom algorithms or software that are central to the research but not yet described in published literature, software must be made available to editors/reviewers. We strongly encourage code deposition in a community repository (e.g. GitHub). See the Nature Research guidelines for submitting code & software for further information.

Data

Policy information about availability of data

All manuscripts must include a data availability statement. This statement should provide the following information, where applicable:

- Accession codes, unique identifiers, or web links for publicly available datasets
- A list of figures that have associated raw data
- A description of any restrictions on data availability

All data available upon reasonable request.

Field-specific reporting

Please select the one below that is the best fit for your research. If you are not sure, read the appropriate sections before making your selection.

[x] Life sciences [ ] Behavioural & social sciences [ ] Ecological, evolutionary & environmental sciences

For a reference copy of the document with all sections, see nature.com/documents/nr-reporting-summary-flat.pdf

Life sciences study design

All studies must disclose on these points even when the disclosure is negative.

Sample size	No statistical methods were used to predetermine the sample size. Sample sizes were estimated based on our preliminary experiments. We made the effort to minimize the sample size of animal studies which was sufficient to reproducibly observe statistically significant difference.
Data exclusions	No data was excluded from our study.
Replication	Experiments were independently repeated two to three times. All attempts to reproduce the results were successful.
Randomization	Mice were assigned at random to treatment groups in all mouse studies. Mice of different groups were maintained mixed (at random) among cages.
Blinding	Adhesion scoring was performed in a blinded manner.

Reporting for specific materials, systems and methods

We require information from authors about some types of materials, experimental systems and methods used in many studies. Here, indicate whether each material, system or method listed is relevant to your study. If you are not sure if a list item applies to your research, read the appropriate section before selecting a response.

Materials & experimental systems

n/a	Involved in the study	
[ ]	[x]	Antibodies
[x]	[ ]	Eukaryotic cell lines
[x]	[ ]	Palaeontology
[ ]	[x]	Animals and other organisms
[x]	[ ]	Human research participants
[x]	[ ]	Clinical data

Methods

n/a	Involved in the study	
[x]	[ ]	ChIP-seq
[ ]	[x]	Flow cytometry
[x]	[ ]	MRI-based neuroimaging

Antibodies

Antibodies used	Antibodies for immunohistochemistry:<br>Anti-F4/80 antibody (clone: BM8), 1:200 dilution (eBioscience, Cat# 14-4801-81)<br>Anti-F4/80 antibody (clone: BM8), biotin conjugated, 1:200 dilution (BioLegend, Cat# 123105)<br>Anti-Fibrin antibody, 1:500 dilution (Abcam, Cat# ab34269)<br>Anti-PDPN antibody (clone: 8.1.1), 1:1000 dilution (eBioscience, Cat# 14-5381-81)<br>Anti-cytokeratin 19 antibody (clone: EP1580Y), 1:500 dilution (Abcam, Cat# ab52625)<br>Anti-mesothelin antibody, 1:100 dilution (Invitrogen, Cat# PA5-79698)<br>Anti-laminin antibody, 1:1000 dilution (Sigma, Cat# L9393)<br>Anti-CD206 antibody (clone: C068C2), 1:200 dilution (BioLegend, Cat# 141702)<br>Anti-CCR2 antibody (clone: SA203G11), Alexa Fluor 647 conjugated, 1:100 dilution (BioLegend, Cat# 150603)<br>Anti-Siglec-F antibody (clone: S17007L), APC conjugated, 1:100 dilution (BioLegend, Cat# 155507)

Anti-Ly6G antibody (clone: 1A8), APC conjugated, 1:100 dilution (BioLegend, Cat# 127613)
Anti-ICAM2 (CD102) antibody (clone: 3C4), Alexa Fluor 647 conjugated, 1:100 dilution (BioLegend, Cat# 105611)
Anti-B220 antibody (clone: RA3-6B2), biotin conjugated, 1:200 dilution (eBioscience, Cat# 13-0452-82)
Anti-CD4 antibody (clone: H129.19), biotin conjugated, 1:200 dilution (BD Biosciences, Cat# 553648)
Anti-CD8a antibody (clone: 53-6.7), 1:200 dilution (BD Biosciences, Cat# 558733)

Antibodies for flow cytometry:
Anti-mouse CD16/CD32 antibody (clone: 93), 1:100 dilution (Invitrogen, Cat# 14-0161-85)
Anti-CD206 antibody (clone: C068C2), Alexa Fluor 488 conjugated, 1:100 dilution (BioLegend, Cat# 141710)
Anti-F4/80 antibody (clone: BM8), PE conjugated, 1:200 dilution (BioLegend, Cat# 123110)
Anti-CD11b antibody (clone: M1/70), APC conjugated, 1:400 dilution (eBioscience, Cat# 17-0112-82)
Anti-CCR2 antibody (clone: SA203G11), Alexa Fluor 647 conjugated, 1:100 dilution (BioLegend, Cat# 150603)
Anti-MHCII (I-Ab) antibody (clone: AF6-120.1), APC conjugated, 1:200 dilution (BioLegend, Cat# 116417)
Anti-ICAM2 (CD102) antibody (clone: 3C4), Alexa Fluor 647 conjugated, 1:200 dilution (BioLegend, Cat# 105611)
Anti-CCR2 antibody (clone: SA203G11), Brilliant Violet 785 conjugated, 1:100 dilution (BioLegend, Cat# 150621)
Anti-CD11b antibody (clone: M1/70), APC/Cyanine7 conjugated, 1:400 dilution (BioLegend, Cat# 101226)
Anti-CSF-1R antibody (clone: AFS98), PE/Cyanine7 conjugated, 1:200 dilution (BioLegend, Cat# 135523)
Anti-Ly6C antibody (clone: HK1.4), Brilliant Violet 605 conjugated, 1:200 dilution (BioLegend, Cat# 128035)
Anti-Siglec-F antibody (clone: S17007L), APC conjugated, 1:100 dilution (BioLegend, Cat# 155507)
Anti-Ly6G antibody (clone: 1A8), Brilliant Violet 510 conjugated, 1:200 dilution (BioLegend, Cat# 127633)
Anti-Ly6G antibody (clone: 1A8), APC conjugated, 1:200 dilution (BioLegend, Cat# 127613)
Anti-B220 antibody (clone: RA3-6B2), APC conjugated, 1:200 dilution (BioLegend, Cat# 103211)
Anti-mouse CD3ε antibody (clone: 145-2C11), APC conjugated, 1:200 dilution (BioLegend, Cat# 100311)
Anti-CD4 antibody (clone: H129.19), PE conjugated, 1:200 dilution (BD Biosciences, Cat# 553652)
Anti-CD8a antibody (clone: 53-6.7), Alexa Fluor 488 conjugated, 1:200 dilution (BioLegend, Cat# 100726)
Rat IgG2a kappa Isotype control antibody, Alexa Fluor 488 conjugated (AbD Serotec, Cat# MCA1124A488)
Rat IgG2a kappa Isotype control antibody (clone: eBR2a), PE conjugated (eBioscience, Cat# 12-4321-41)
Rat IgG2a kappa Isotype control antibody (clone: RTK2758), APC conjugated (BioLegend, Cat# 400511)
Rat IgG2b kappa Isotype control antibody (clone: eB149/10H5), APC conjugated (eBioscience, Cat# 17-4031-81)
Rat IgG2a kappa Isotype control antibody (clone: RTK2758), Alexa Fluor 647 conjugated (BioLegend, Cat# 400526)
Rat IgG2b kappa Isotype control antibody (clone: RTK4530), Alexa Fluor 647 conjugated (BioLegend, Cat# 400626)
Rat IgG2b kappa Isotype control antibody (clone: RTK4530), Brilliant Violet 785 conjugated (BioLegend, Cat# 400647)
Rat IgG2b kappa Isotype control antibody (clone: RTK4530), APC/Cyanine7 conjugated (BioLegend, Cat# 400623)
Rat IgG2a kappa Isotype control antibody (clone: RTK2758), PE/Cyanine7 conjugated (BioLegend, Cat# 400521)
Rat IgG2c kappa Isotype control antibody (clone: RTK4174), Brilliant Violet 605 conjugated (BioLegend, Cat# 400727)
Rat IgG2a kappa Isotype control antibody (clone: RTK2758), Brilliant Violet 510 conjugated (BioLegend, Cat# 400547)

Antibodies for Proximity ligation assay:
Anti-CD11b antibody (clone: M1/70), 1:200 dilution (eBioscience, Cat# 14-0112-85)
Anti-Fibrin antibody, 1:500 dilution (Abcam, Cat# ab34269)
Goat anti-Rat IgG antibody, AlexaFluor 488 conjugated, 1:300 dilution (Invitrogen, Cat# A11006)
Anti-F4/80 antibody (clone: BM8), APC conjugated, 1:200 dilution (BioLegend, Cat# 123116)
Goat anti-Rabbit IgG antibody, AlexaFluor 405 conjugated, 1:300 dilution (Invitrogen, Cat# A31556)
Anti-F4/80 antibody (clone: BM8), 1:200 dilution (eBioscience, Cat# 14-4801-81)
Anti-CD11b antibody (clone: M1/70), APC conjugated, 1:200 dilution (eBioscience, Cat# 17-0112-82)

Antibodies for in vivo treatment:
Anti-CD11b antibody (clone: 5C6) (Invitrogen, Cat# MA5-16528)
Rat IgG2b control antibody (clone: eB149/10H5) (eBioscience, Cat# 16-4031-85)

Validation

All antibodies are commercially available. These validation information are provided on the manufacturer's websites.

Animals and other organisms

Policy information about studies involving animals; ARRIVE guidelines recommended for reporting animal research

Laboratory animals

Ten- to twelve-week-old male C57BL/6 mice were obtained from the Jackson Laboratory. All mice were maintained under pathogen-free conditions.

Wild animals

N/A

Field-collected samples

N/A

Ethics oversight

All animal studies were performed with the approval of the ethics committee of the Queen Mary University of London and the UK Home Office (project license PPL70/8503). More detailed information are described in methods in the "Study approval" section.

Note that full information on the approval of the study protocol must also be provided in the manuscript.

Flow Cytometry

Plots

Confirm that:

- [x] The axis labels state the marker and fluorochrome used (e.g. CD4-FITC).
- [x] The axis scales are clearly visible. Include numbers along axes only for bottom left plot of group (a 'group' is an analysis of identical markers).
- [x] All plots are contour plots with outliers or pseudocolor plots.
- [x] A numerical value for number of cells or percentage (with statistics) is provided.

Methodology

Sample preparation	Sample preparation is described in methods in the "Peritoneal cell isolation and flow cytometry analysis" section.
Instrument	All samples were read on BD LSRFortessa cell analyzer (BD Biosciences).
Software	All data were analyzed with FlowJo software version 10 (Tree Star).
Cell population abundance	N/A
Gating strategy	Forward versus side scatter (FSC-A vs SSC-A) gating was used to identify cells of interest and exclude debris. Singlets were gated according to the pattern of FSC-A vs FSC-H, followed by SSC-H vs SSC-W. Dead cells were excluded by DAPI staining. Positive populations were determined by the specific antibodies, which were distinct from negative populations. Further gating strategies will be provided upon reasonable request.

- [x] Tick this box to confirm that a figure exemplifying the gating strategy is provided in the Supplementary Information.
